# Comparative Evaluation of Drug Incorporation and Release Profiles in Six Specific Porous Vaterite CaCO_3_ Formulations

**DOI:** 10.3390/ph19091446

**Published:** 2026-09-12

**Authors:** Nawar Mikhael Toma, Lubna Abdalkarim Sabri, Duaa Jaafar Jaber Al-Tamimi, Sajad Moneim Aljubouri, Shahbaa Mohammed Ati

**Affiliations:** 1 Department of Pharmaceutics, College of Pharmacy, University of Baghdad, Baghdad 10047, Iraq; lobna.sabri@copharm.uobaghdad.edu.iq (L.A.S.); shahbaa.m@copharm.uobaghdad.edu.iq (S.M.A.); 2 Department of Pharmaceutics, College of Pharmacy, Madenat Alelem University, Baghdad 10006, Iraq; duaa.jaafar@mauc.edu.iq; 3 Department of Pharmacology, College of Medicine, Ibn Sina University for Medical and Pharmaceutical Sciences, Baghdad 10012, Iraq; sajad.m.aljubouri@ibnsina.edu.iq

**Keywords:** vaterite calcium carbonate, PVP stabilization, encapsulation efficiency, controlled drug release, metformin, meloxicam

## Abstract

**Background:** Vaterite calcium carbonate (CaCO_3_) particles are promising pH-sensitive carriers for oral drug delivery, but the influence of drug hydrophilicity on loading and release is still not well defined. In this study, we evaluate the encapsulation and release profiles of six specific formulations utilizing metformin and meloxicam across distinct CaCO_3_ carrier variants and loading methodologies. **Methods:** We prepared vaterite CaCO_3_ particles as native (and PVP-stabilized) carriers and utilized co-precipitation, post-loading (adsorption), and sonication-assisted loading techniques. We subsequently evaluated carrier morphology, phase stability, encapsulation efficiency, surface adsorption capacity, and in vitro drug release in simulated gastric (pH 1.2) and intestinal (pH 6.8) media. **Results:** Quantitative image analysis confirmed that porous vaterite particles formed primarily in the micron range, with PVP stabilization significantly reducing the mean particle diameter compared to native carriers (0.966 µm versus 1.189 µm). Furthermore, extended exposure to aqueous media induced partial transformation to calcite. The highest encapsulation efficiency for metformin (93.0 ± 1.5%) was achieved using co-precipitation, whereas for meloxicam (79.6 ± 1.9%), it was optimized through sonication-assisted adsorption on the PVP-modified carriers. In vitro release showed a clear pH-dependent behavior; the fast dissolution of the vaterite through gastric pH was associated with the fast release of metformin, especially in the calcite-containing formulation that produced the greatest release (98.0 ± 3.3%) in 15 min. At intestinal pH, metformin and meloxicam exhibited sustained release as in the Korsmeyer–Peppas model. **Conclusions:** The evaluated carrier compositions and loading methodologies distinctly influenced the encapsulation and release profiles of the six prepared formulations. These specific formulation comparisons highlight practical considerations for utilizing vaterite carriers for varied release targets.

## 1. Introduction

The delivery of drugs for chronic disease management is a major challenge. Traditional pharmacotherapy requires long dosing, and drug concentrations vary widely at target sites, which can lead to lower effectiveness or toxicity. Therefore, well-controlled delivery systems that keep the drug in a steady state while reducing dosing are beneficial to long-term outcomes [1].

Despite the development of polymeric nanoparticles, liposomes, and micellar systems, there are still limitations such as burst release, low drug-loading efficiency, limited co-encapsulation of hydrophilic and hydrophobic drug molecules [2], cytotoxicity, biodegradation, and clearance problems [3]. The above challenges have motivated the development of more versatile carriers for drug delivery and safety at the same time [4,5].

Calcium carbonate (CaCO_3_) is an attractive carrier due to its biocompatibility, biodegradability, low cost, and tunability [6,7]. CaCO_3_ can be found in a variety of polymorphs—stable calcite, metastable vaterite, and others—and vaterite is a good carrier due to its mesoporous structure and high surface area, thus enabling a better space for drug delivery. Vaterite can accept hydrophilic and hydrophobic molecules, and it can be pH-responsive under acidic or endosomal conditions for controlled (or site-selective) release [6,7,8].

Most of the research is on nanoscale vaterite (small vaterite under 500 nm) [9], although micron- and submicron-sized particles are not as well studied despite their scalability and manufacturing advantages [10]. It is difficult to obtain a reproducible, phase-pure vaterite, as its polymer production is highly dependent on the chemical factors involved (precursors, pH, additives), and vaterite can change to more stable forms (calcite is stable), thereby lowering porosity and control over loading and release mechanisms [6,7,11]. To overcome this instability, polymeric additives such as polyvinylpyrrolidone (PVP) are often employed as crystal growth modifiers [12]. The steric stabilization of PVP prevents the unconstrained nucleation and growth of CaCO_3_ and hence prevents the premature transformation of metastable vaterite into calcite [13,14]. This polymer-assisted stabilization is crucial for ensuring the highly porous microarchitecture needed for loading and release of drugs.

Moreover, many studies have examined single-drug systems but are not clear on the role of drug physicochemical properties in loading and release of vaterite carriers [6,7]. To address this, we compare the loading and pH-responsive release of two drugs with different solubility profiles. Metformin hydrochloride is a highly water-soluble and hydrophilic drug for type 2 diabetes [15], and it is required for long-term administration to minimize dosing frequency and gastrointestinal side effects [16,17]. On the other hand, meloxicam is a highly lipophilic, poorly water-soluble non-steroidal anti-inflammatory drug (NSAID) for chronic pain that can benefit from carrier systems that enable it to dissolve and release across the gastrointestinal pH gradient [18]. To clarify how drug hydrophilicity and carrier modification influence encapsulation efficiency and site-specific release, we investigate three different loading strategies: rapid co-precipitation, sonication-assisted adsorption, and prolonged post-synthetic adsorption. Ultimately, evaluating these six distinct formulations provides comparative data to guide the selection of specific vaterite carrier preparations for either rapid gastric or sustained intestinal drug delivery.

## 2. Results and Discussion

### 2.1. Morphological, Interfacial, and Surface Characterization of Blank Carriers and Drug-Loaded Formulations

**Figure 1:**
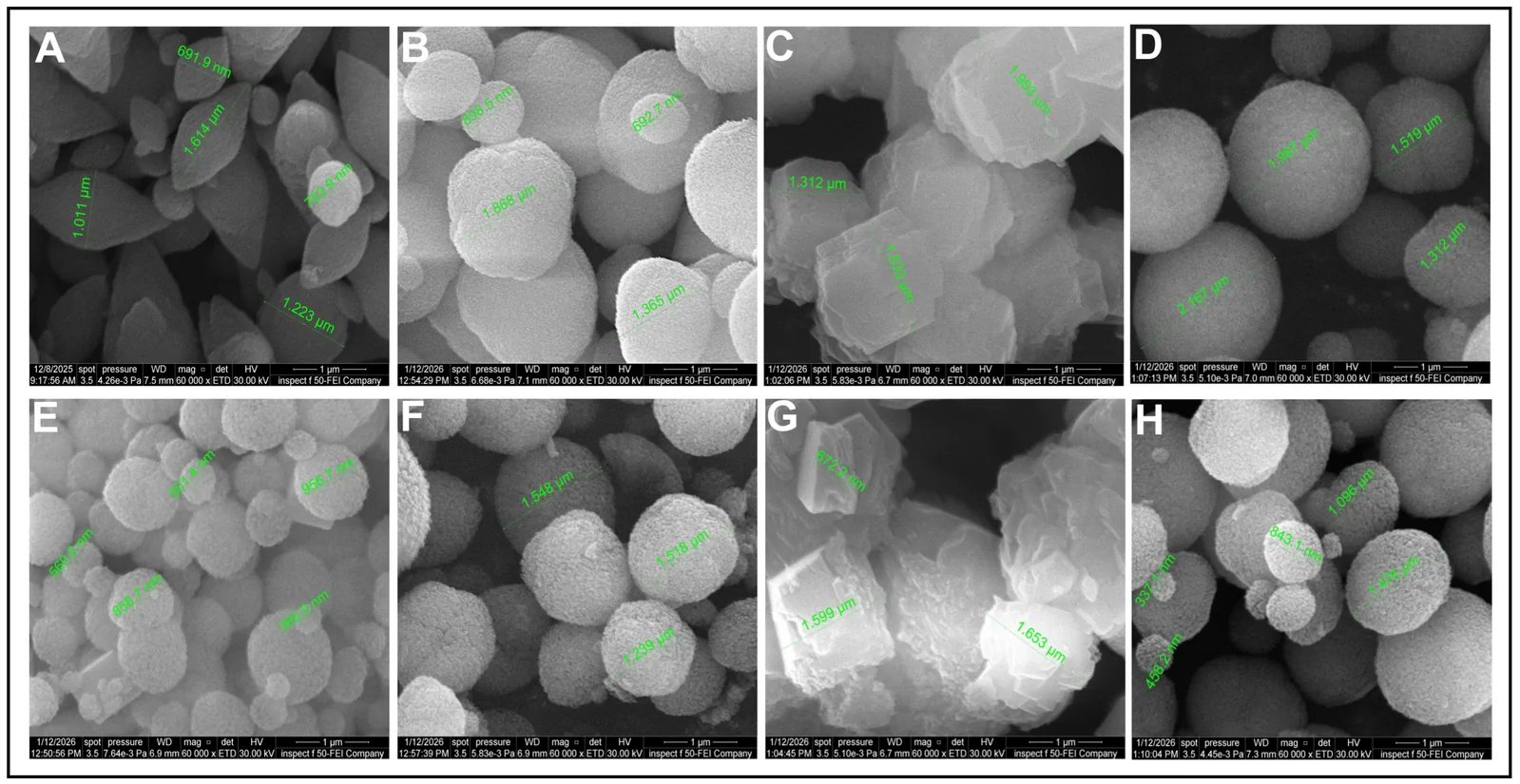
FE-SEM micrographs of the blank carriers and drug-loaded CaCO_3_ formulations: (**A**) Carrier A, (**B**) Formula F1, (**C**) Formula F3, (**D**) Formula F5, (**E**) Carrier B, (**F**) Formula F2, (**G**) Formula F4, and (**H**) Formula F6. Panels (**A**,**E**) show the morphology of the blank CaCO_3_ carriers A and B, which exhibit scalenohedral and spherical structures, respectively. Panels (**A**) through (**H**) illustrate the surface morphologies and particle dimensions of the various drug-loaded CaCO_3_ formulations, with F1, F2, F5, and F6 predominantly showing spherical particles, while F3 and F4 present distinct rhombohedral or cubic structures.

FE-SEM was used to study the morphology of the newly synthesized calcium carbonate carriers before and after drug loading (Figure 1). The clear differences in shape and surface properties between Carrier A and Carrier B clearly show the role of PVP in crystal growth [6,13]. When CaCl_2_ and Na_2_CO_3_ are mixed very quickly, the calcium carbonate is nucleated as soon as the medium reaches a certain level of supersaturation. In the unmodified system (Carrier A), the nuclei are consolidated and grow without constraint, resulting in irregular particles. As vaterite is the least stable CaCO_3_ polymorph [13,14], unconstrained nucleation and growth in this system can easily lead to irregular particles, even if the bulk phase is vaterite.

In comparison to Carrier A, the surface of Carrier B features a rough, granular layer that represents a more organized, porous structure. Furthermore, number-weighted size distribution analysis of the FE-SEM micrographs using Fiji revealed a distinct reduction in particle dimensions due to the steric stabilization of PVP. Carrier A exhibited a mean particle diameter of 1.189 ± 0.281 µm, whereas Carrier B displayed a significantly smaller size distribution with a mean diameter of 0.966 ± 0.188 µm. Following drug loading, mean particle dimensions varied based on the formulation strategy, spanning from 1.359 ± 0.165 µm for F6 to 2.138 ± 0.14 µm for F5. This initial size reduction in the blank carrier increases the available surface area, directly contributing to the enhanced adsorption capacity observed in the PVP-modified formulations. This is due to the steric stabilization of PVP: the polar carbonyl and pyrrolidone groups along its backbone interact with surface Ca^2+^ ions on newly formed CaCO_3_ nuclei [13]. This adsorbed layer prevents particle aggregation and growth, yielding small, uniform spherical vaterite microparticles, similar to previous work on the polymer-mediated control of CaCO_3_ crystallization [13,14].

In F1 and F2, the parent carrier was still spherical and porous and therefore only showed small aggregation and minor surface roughening, indicating that the structure of the carrier was not distorted by rapid drug loading. The F3 and F4 formulations were formed after prolonged adsorption in the presence of the drug but showed severe structural degradation, and thus the porous spherical structure did not hold firm. The crystal structure of the parent carrier was changed to a denser crystalline form after long exposure to aqueous conditions [6,7,19]. PVP-modified versions (F5 and F6) demonstrated the highest structural stability and had a well-defined spherical shape with very low collapse. In general, the FE-SEM results indicate that fast loading and PVP modification can preserve the porous carrier structure, whereas prolonged processing leads to the deterioration of the structure, which is commonly observed in solvent-mediated vaterite-to-calcite transition studies [6,7,19].

Methylene blue adsorption capacity was higher in Carrier B (248.7 ± 1.15 mg/g) compared to Carrier A (228.4 ± 0.3 mg/g). As methylene blue is a cationic dye, this metric reflects both electrostatic surface availability and structural features; however, it does not provide absolute pore volume data. This is consistent with the FE-SEM results, where the PVP modification produced more uniform, rougher-textured particles, likely due to the higher accessibility of adsorption sites after the crystallization of the PVP [13,20].

### 2.2. Zeta Potential Analysis

**Figure 2:**
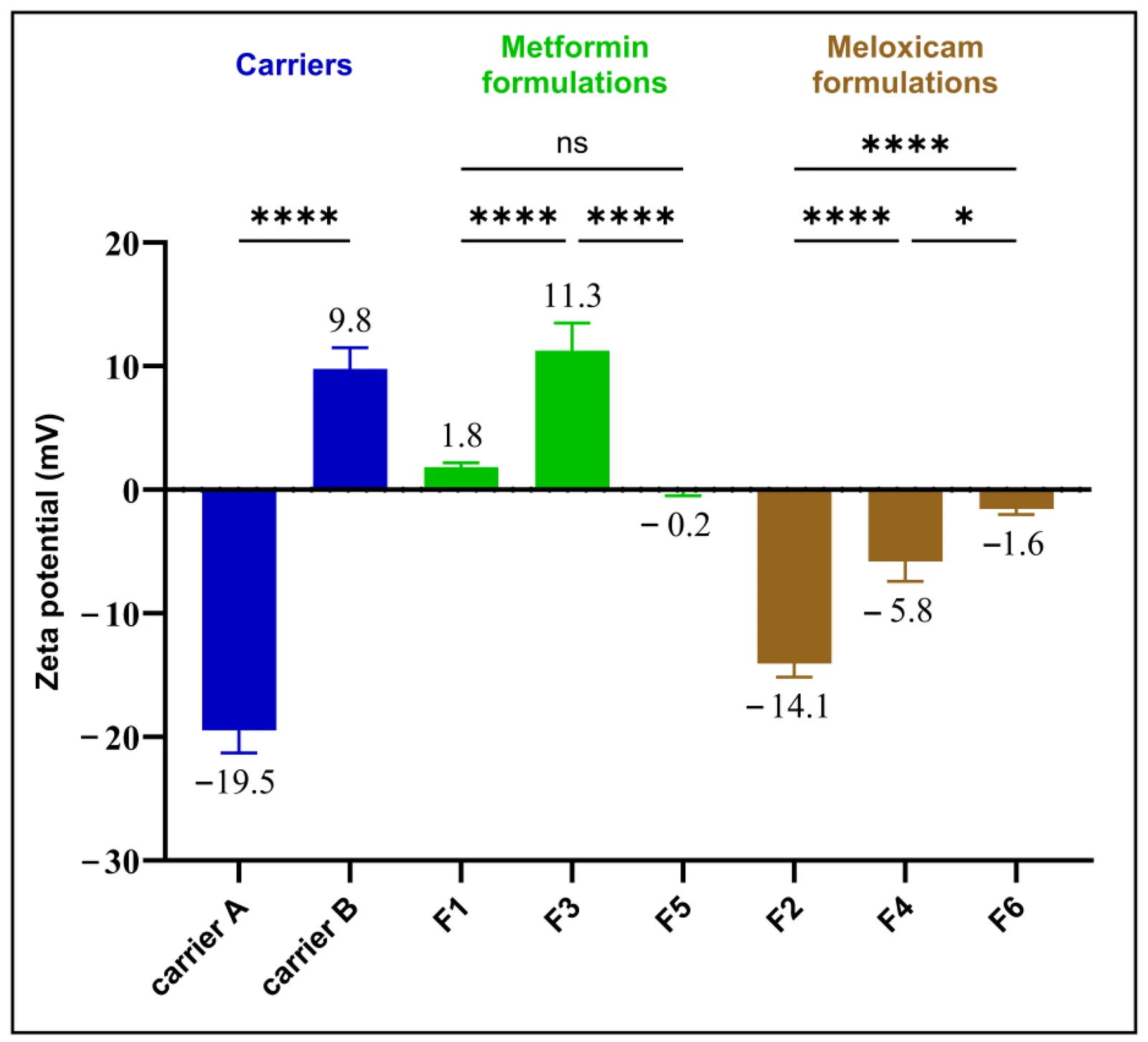
Zeta potential of the blank carriers and drug-loaded CaCO_3_ formulations. Data are presented as mean ± SD (*n* = 3). Statistical analysis was carried out using one-way ANOVA followed by Tukey’s *post hoc* test. “ns” Indicates no significant difference, “*” indicates *p*-value < 0.05, “****” indicates *p*-value < 0.0001.

The zeta potential measurements of the combined carrier composition and drug loading show a significant change in the electrostatic properties of the formulations (Figure 2). The unloaded Carrier A has a zeta potential of −19.47 ± 1.82 mV, whereas the carrier B has a less negative value (−9.77 ± 1.71 mV; *p* < 0.05). This reduction in surface charge magnitude is probably because the nonionic PVP chains adsorbed onto the carrier surface could partially shield the exposed carbonate groups and shift the electrokinetic shear plane, i.e., reduce the surface potential [13,21].

In contrast, depending on the drug content of the formulation the zeta potential changes with drug loading. For the metformin-loaded formulation the zeta potential value is much higher and the highest positive value is F3 at an average of 11.25 ± 2.24 mV, which is much higher than F1 and F5. This very strong positive shift is also in line with the fact that metformin is monoprotonated and positively charged in the near-neutral state of the particle surface and thus has electrostatic bond with the surface of the particle after adsorption from the synthetic surface [22].

The most of the meloxicam-loaded formulation remained negative zeta potentials, as the average for the carriers were as for meloxicam-loaded carriers [23], but the gradient towards charge neutrality was observed in the order F2 < F4 < F6. There were clear differences among the formulations (Figure 2). The lower zeta potential of F6 suggests that the loading of PVP has not significantly affected the surface electrostatic properties of the carrier in the loading of the drug, but might be masking the surface charge.

Although the zeta potential of all the formulations was not more than ±30 mV (the usual value for electrostatically driven colloidal stable state), PVP may still have some steric action but not as much as the surface charge, as in the case of DP [13,21]. Overall, the composition of the carriers and drug loading strategies demonstrably altered the electrokinetics of the formulations in deionized water, confirming successful PVP modification and surface-level drug incorporation. However, because these measurements were not performed in high-ionic-strength buffered media, they serve solely as formulation characterization metrics and should not be extrapolated to predict electrostatic drug-release behavior at physiological pH.

### 2.3. Solid-State Characterization

**Figure 3:**
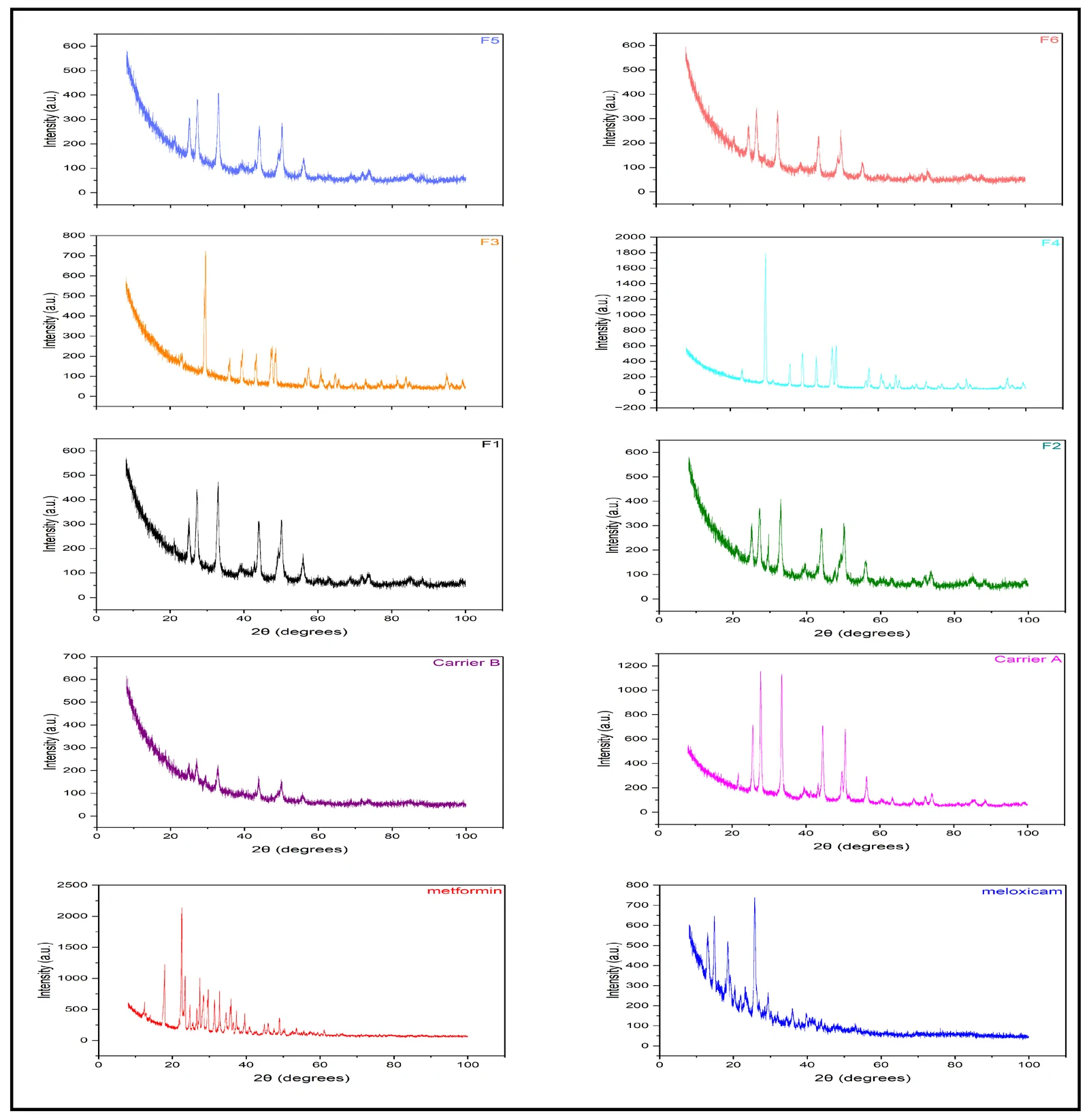
PXRD patterns of the blank carriers and drug-loaded formulations.

To study the crystalline phase composition, molecular interactions, and physical state of the incorporated drugs, the developed formulations were characterized using PXRD, FTIR, and DSC. PXRD was used to identify the calcium carbonate polymorphs and to evaluate the crystallinity changes. FTIR was used to detect the possible interactions between the carrier and the drugs. DSC was used to study the thermal behaviour and physical state of the drugs. The PXRD pattern of the blank carriers (A and B), metformin-loaded formulations (F1, F3, and F5), and meloxicam-loaded formulations (F2, F4, and F6) were shown in Figure 3.

The PXRD diffractogram of Carrier A has characteristic reflections of about 2θ = 24.9°, 27.0°, and 32.8°, which further showed that the crystalline polymorphs of CaCO_3_ were mostly composed of vaterite. The same vaterite diffraction pattern is shown for Carrier B, which suggests that the incorporation of PVP did not change the crystalline phase composition of the carrier. However, we did observe a slight change in the peak positions and the relative peak intensities, which could be due to the interaction of PVP with the crystals. Such an effect has been observed previously for polymer-assisted CaCO_3_ precipitation systems, with PVP adsorbing on crystal surfaces, inhibiting crystal growth, and stabilizing the metastable vaterite phase [6,13].

The loading process had a strong impact on the carrier structure. The F1, F2, F5, and F6 formulations produced with fast loading in this study were able to retain the typical vaterite reflections and hence the original carrier structure when the drug was introduced. The calcite structure was minimal in F2 and F6, and the diffraction peak was weak around 2θ = 29.5°. In the case of formulations F3 and F4, the vaterite reflections disappeared, and a peak in the diffraction at 2θ = 29.4° was present, which is typical for calcite. Therefore, it is clear that the long process of aqueous processing leads to the transformation of metastable vaterite into thermodynamically stable calcite, and this is known to happen for CaCO_3_ systems in the presence of water [6,7,19].

The crystalline peaks of pure metformin (2θ = 17.7° and 22.5°) [24] and meloxicam (2θ = 25.7°) [25] in all the drug-loaded formulations (see Figure 3) were not observable. If these reflections are absent, the drugs are either dispersed in the carrier matrix (in the amorphous or low crystallinity state) or present at concentrations below the PXRD detection limit. The porous nature of the vaterite particles and the large size of the surface area of the vaterite molecules could help to contain the drugs in the carrier pores and prevent them from growing in size and shape. Similar changes in drug crystallinity after incorporation into porous inorganic carriers have been reported for several drug-delivery systems and are generally associated with drug dispersion and better release performance [6,26].

**Figure 4:**
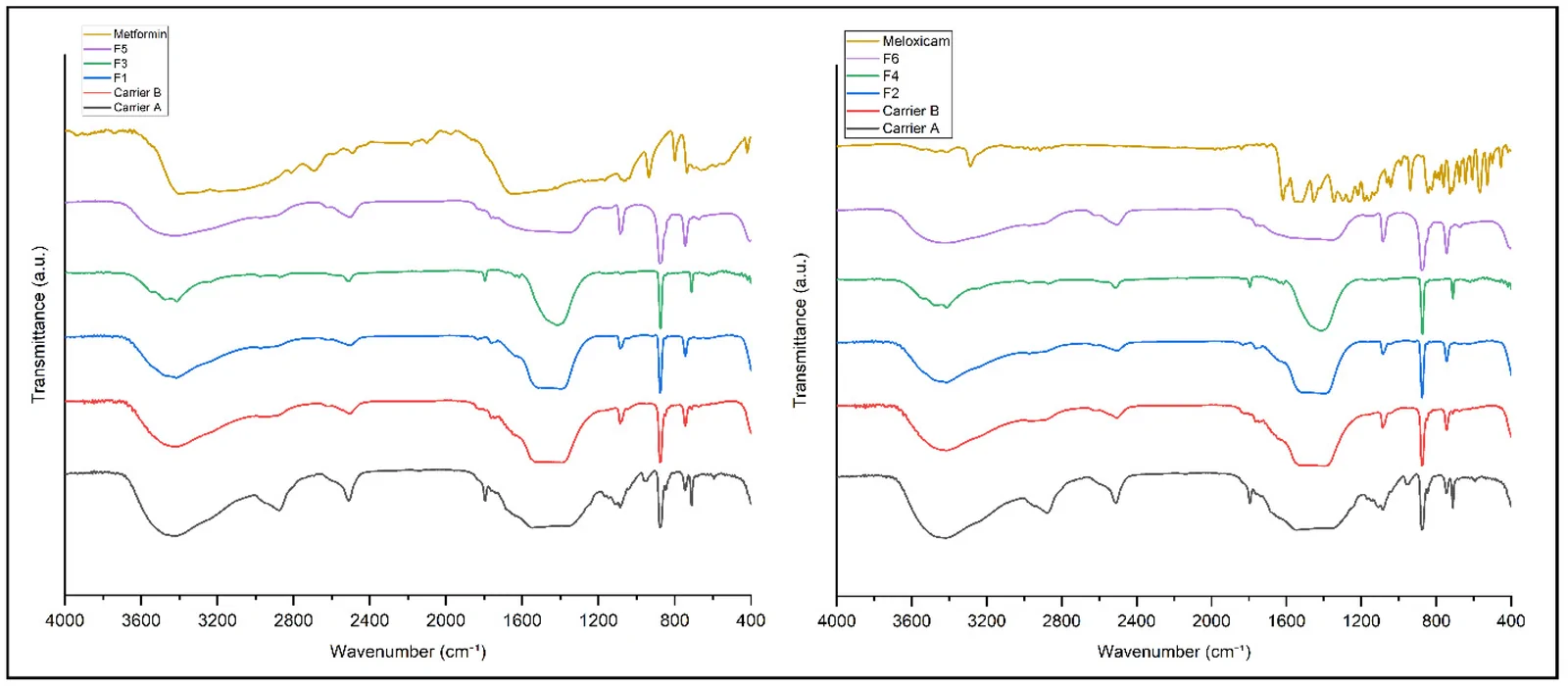
FTIR spectra of the blank carriers, pure drugs, and drug-loaded formulations.

Overall, the PXRD results show that PVP was used to stabilize the vaterite-rich carrier structure and the loading method was important for carrier phase stability. Fast loading significantly preserved the original carrier structure, and drug incorporation was done successfully without crystalline drug deposition. FTIR spectroscopy was performed to determine the chemical composition of the blank carriers and drug-loaded formulations and for the possible interaction of CaCO_3_, PVP, and drugs (Figure 4).

Carrier A showed the characteristic bands of carbonate absorption at about 711, 744, 874 and 1086 cm^−1^. The presence of bands at 711 and 744 cm^−1^ together with the high absorption at 1086 cm^−1^ are consistent with carbonate concentrations for vaterite-rich CaCO_3_ systems [27] and support the PXRD determination that vaterite is the dominant crystalline phase.

Carrier B retained carbonate bands but showed an abrupt shift in band position and intensity in the carbonate vibration region [13], which suggests that PVP has altered the environment at the CaCO_3_ surface. This is predicted to be a function of the coordination between PVP’s carbonyl groups and the surface calcium ions, which adsorbs polymer on the surface [13,28].

The FTIR spectra of formulations F1, F2, F5, and F6 remained largely comparable to those of the parent carriers, indicating that drug loading did not significantly alter the carbonate framework. In contrast, F3 and F4 showed spectra dominated by the carbonate band near 711 cm^−1^, with reduced intensity of vaterite-associated absorptions, consistent with transformation toward the calcite polymorph following prolonged aqueous processing [6,7,29].

Characteristic functional group vibrations of metformin and meloxicam remained detectable in the corresponding formulations, despite partial overlap with the intense carbonate absorptions. No new absorption bands or spectral shifts indicative of chemical bond formation were observed, indicating that drug incorporation occurred without chemical degradation and that drug–carrier interactions were predominantly physical, consistent with adsorption, pore confinement, and hydrogen bonding as the dominant mechanisms reported for porous CaCO_3_-based drug carriers [6,30]. Overall, the FTIR results confirm the chemical compatibility of metformin and meloxicam with the developed carrier systems.

**Figure 5:**
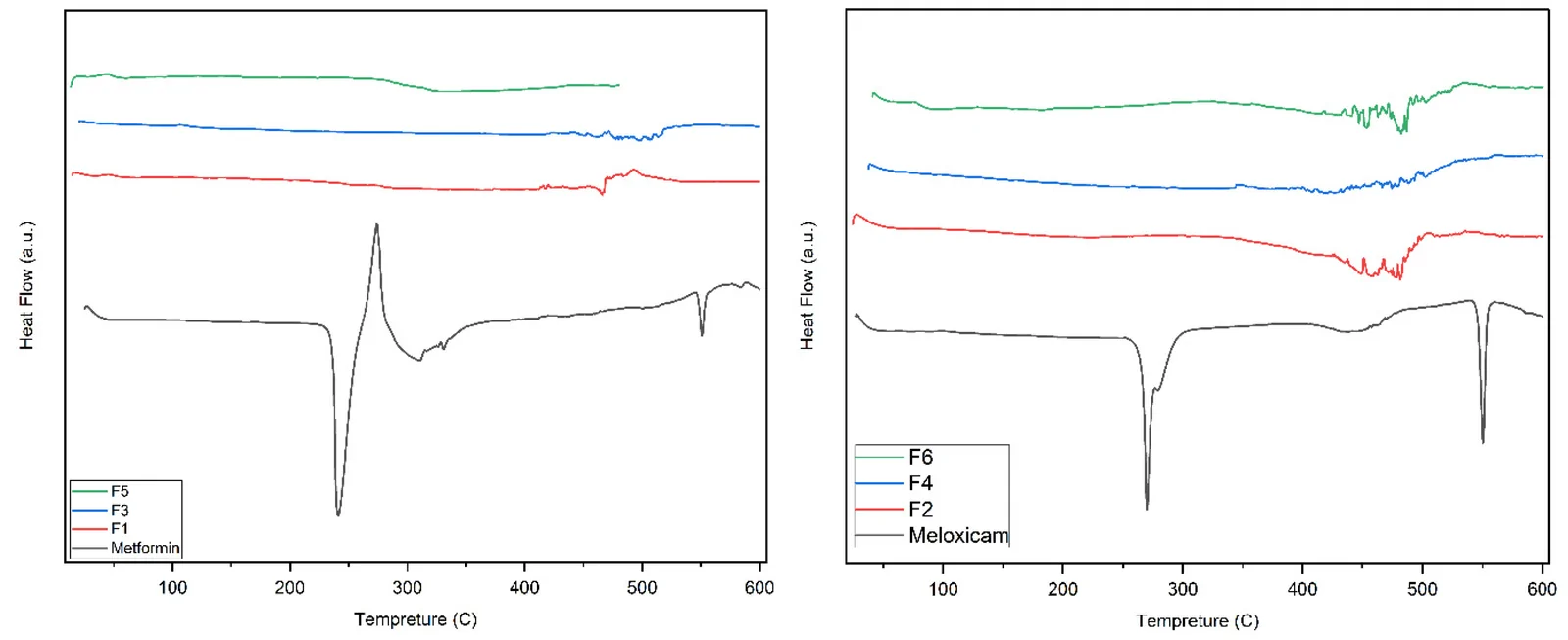
DSC thermograms of the pure drugs and corresponding drug-loaded formulations.

Differential scanning calorimetry was performed to evaluate the thermal behavior of the pure drugs and drug-loaded formulations and to further elucidate the physical state of the incorporated drugs (Figure 5).

Pure metformin exhibits a characteristic endothermic melting at ~ 230–250 °C, which means that it is crystalline [16]. This thermal transition was not observed in F1, F3, and F5. Similarly, pure meloxicam exhibits a sharp endothermic melting peak at ~ 260–270 °C [18]. This melting peak was not observed in F2, F4, and F6. The disappearance of these characteristic melting endotherms suggests that when the drug is added to the CaCO_3_ carrier system it is no longer crystalline.

The absence of drug melting peaks does not mean that the drug is completely amorphized and any remaining crystalline part is not detectable with DSC. These results are in agreement with the PXRD results where no crystalline reflections of both drugs were observed in all formulations.

The porous structure of vaterite CaCO_3_ is likely to have played a role in this phenomenon by restricting the drug molecules in the pores and adsorption sites in such a way that they were not able to move around much and crystalline growth was inhibited [31]. Drug crystallinity decreases are also commonly reported for drugs in mesoporous and porous inorganic carriers and are associated with more efficient dissolution and release [26,30]. No additional thermal events due to drug degradation, chemical incompatibility, or new solid-phase formation were observed for any formulation.

Taken together, the DSC, PXRD, and FTIR results give us solid evidence for the successful incorporation of metformin and meloxicam into these systems: although the vaterite-rich structure is preserved in the rapidly processed formulations, the drug crystallinity is reduced and the carrier is not compromised to any degree. These solid-state modifications might contribute to the improved encapsulation efficiency and release performance.

### 2.4. Encapsulation Efficiency, Dissolution Profile, and Release Kinetics

**Figure 6:**
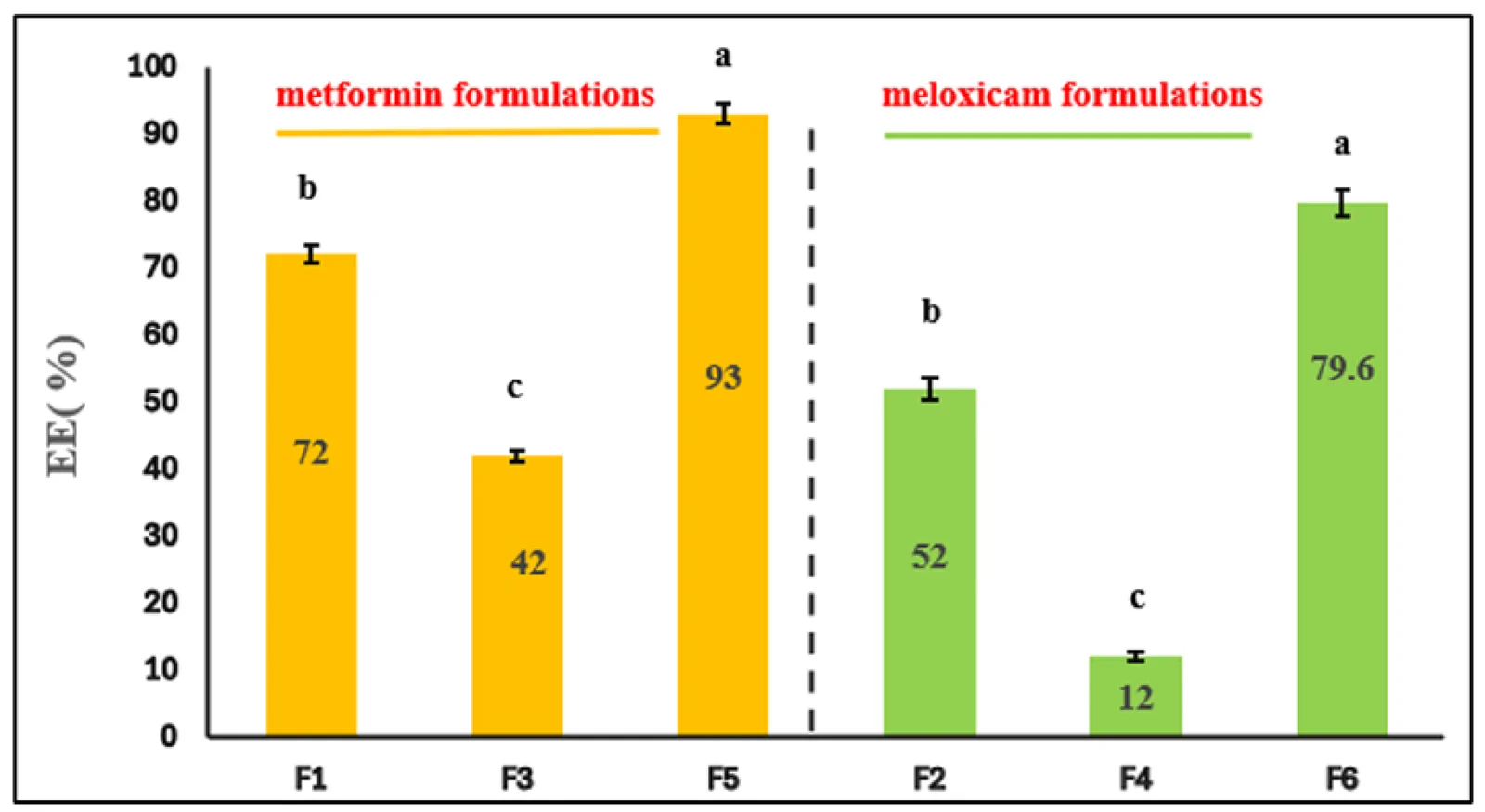
Encapsulation efficiency of the developed metformin- and meloxicam-loaded CaCO_3_ formulations. Data are presented as mean ± SD. Different lowercase letters (a–c) above the bars indicate statistically significant differences between the formulations (*p* < 0.05), as determined by one-way ANOVA followed by Tukey's post-hoc test.

Encapsulation efficiency (EE%) demonstrated the relative success of the loading methodologies when analyzed within each specific drug group (Figure 6). Because the initial drug feeding ratios differed (25 mg for metformin versus 10 mg for meloxicam) and carrier mass is formed in situ during co-precipitation, these EE% values serve as intra-drug comparative metrics rather than absolute cross-drug values. One-way ANOVA followed by Tukey’s post hoc test revealed significant differences among all formulations within both the metformin-loaded and meloxicam-loaded groups (*p* < 0.05).

Among the metformin-loaded formulations, F5 exhibited the highest encapsulation efficiency (93.0 ± 1.5%), followed by F1 (72.0 ± 1.4%), while F3 showed the lowest value (42.0 ± 0.8%). Analyzing the meloxicam-loaded formulations independently reveals a similar internal trend, where F6 achieved the highest relative EE% (79.6 ± 1.9%), followed by F2 (52.0 ± 1.7%), while F4 exhibited the lowest efficiency (12.0 ± 0.6%) for this specific lipophilic drug.

The poorest loading performance was observed for F3 and F4, prepared using extended post-synthetic adsorption. FE-SEM revealed a major deterioration of the original porous architecture and PXRD and FTIR showed that the porous vaterite carrier was altered by the calcite polymorph with the long loading process. Since calcite is less porous and can be accessible to drug less easily than vaterite, this change in the porous vaterite structure may lead to less loading sites and lower drug entrapment efficiency. This is consistent with previous published findings that vaterite and calcite transformation leads to less loading in porous CaCO_3_ drug delivery systems [7]. This relatively low EE% for F4 suggests that morphological deterioration and the transition to a denser calcite phase were the major factors contributing to drug entrapment failure. The vast differences in EE% across formulations (42–93%) compared to the modest difference in baseline MB adsorption (~8.9%) highlight that the active loading mechanism (e.g., direct matrix entrapment during co-precipitation versus reliance on surface adsorption) and carrier phase stability dictate overall encapsulation far more than the baseline adsorption capacity of the starting carrier [31].

The best encapsulation efficiency was achieved with PVP-modified formulations F5 and F6 in comparison with Carrier B because of its significantly better adsorption capacity (248.7 ± 1.15 mg/g) than that of Carrier A (228.4 ± 0.3 mg/g). The structural characterization of the porous vaterite-rich carrier was enhanced by PVP incorporation and FE-SEM revealed a more uniform, well-structured, and structurally stable particle architecture; zeta potential analysis confirmed that PVP modification successfully altered the electrokinetic profile of the carrier, shielding exposed carbonate groups and reducing the magnitude of the negative surface charge. This structural and surface stabilization likely facilitated better drug-carrier interactions during loading, consistent with improved drug transport in polymer-assisted porous CaCO_3_ carriers [32]. In general, within their respective drug categories, the porous vaterite structure was better preserved by the rapid loading strategies and PVP-mediated stabilization, thereby optimizing the relative drug loading efficiency compared to the prolonged post-synthetic adsorption method.

### 2.5. In Vitro Drug Release Behavior

**Figure 7:**
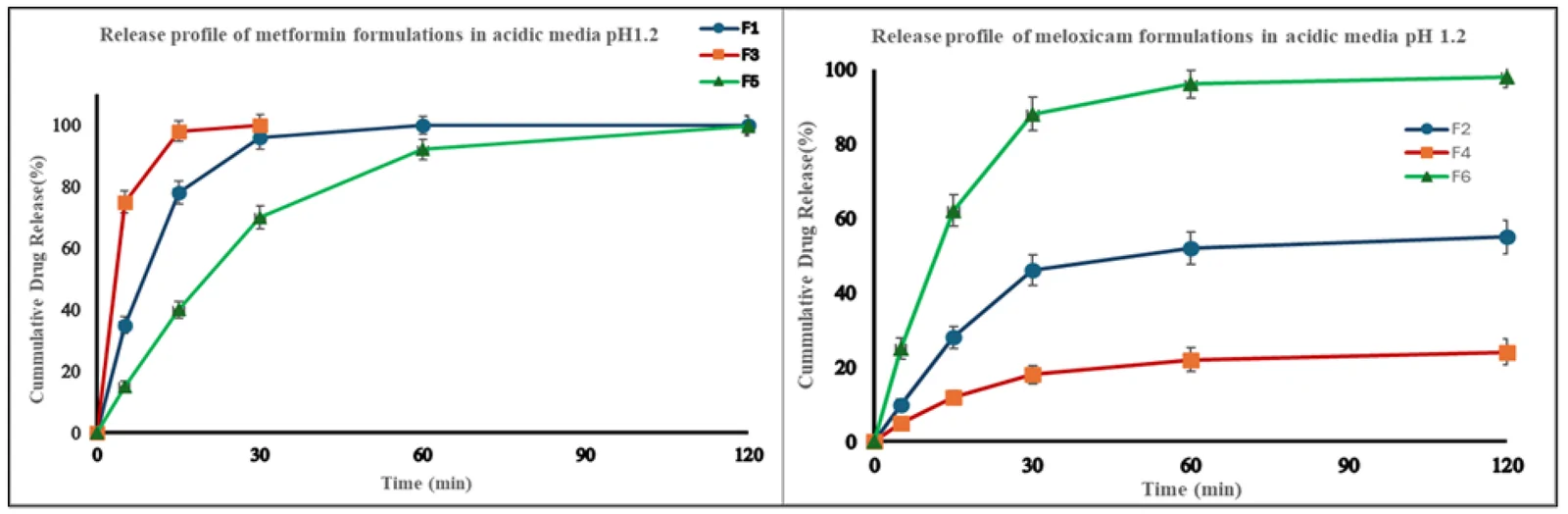
In vitro drug release profiles of metformin- and meloxicam-loaded formulations under simulated gastric (pH 1.2). Data are presented as mean ± SD (*n* = 3). Statistical comparison of the formulations was performed at 15 min using one-way ANOVA followed by Tukey’s post hoc test.

The in vitro release profiles of metformin-loaded formulations (F1, F3, F5) and meloxicam-loaded formulations (F2, F4, F6) have been studied in simulated gastric (pH 1.2) and intestinal (pH 6.8) media as they are the physiological pH gradient along the human gastrointestinal tract. The release behavior is not the same in all the formulations; the carrier composition, structural stability, and drug-loading methodology are significantly different from each other. The release profiles of metformin and meloxicam are shown in Figure 7 in simulated gastric media (pH 1.2).

At 15 min metformin release is the most different in all the formulations (F(2,6) = 234.17, *p* < 0.001). F3 released the most (98.0 ± 3.3%), followed by F1 (78.0 ± 3.8%), and F5 (40.0 ± 2.8%). The rapid release of F3 is expected since it is composed of a lower porosity and has undergone a large calcite transformation in its structure so that a large fraction of the drug was probably being released on the external carrier surface and hence can be dissolved straight away and that is in line with the burst release process in vaterite when this is recovered from a recrystallization and becomes a porous matrix [7]. The slower release of F1 indicates that metformin was only released in part in the porous vaterite matrix when the vaterite was co-precipitated and the lower release of F5 may be the most effective drug retention in the PVP-modified carrier (Figure 7).

**Figure 8:**
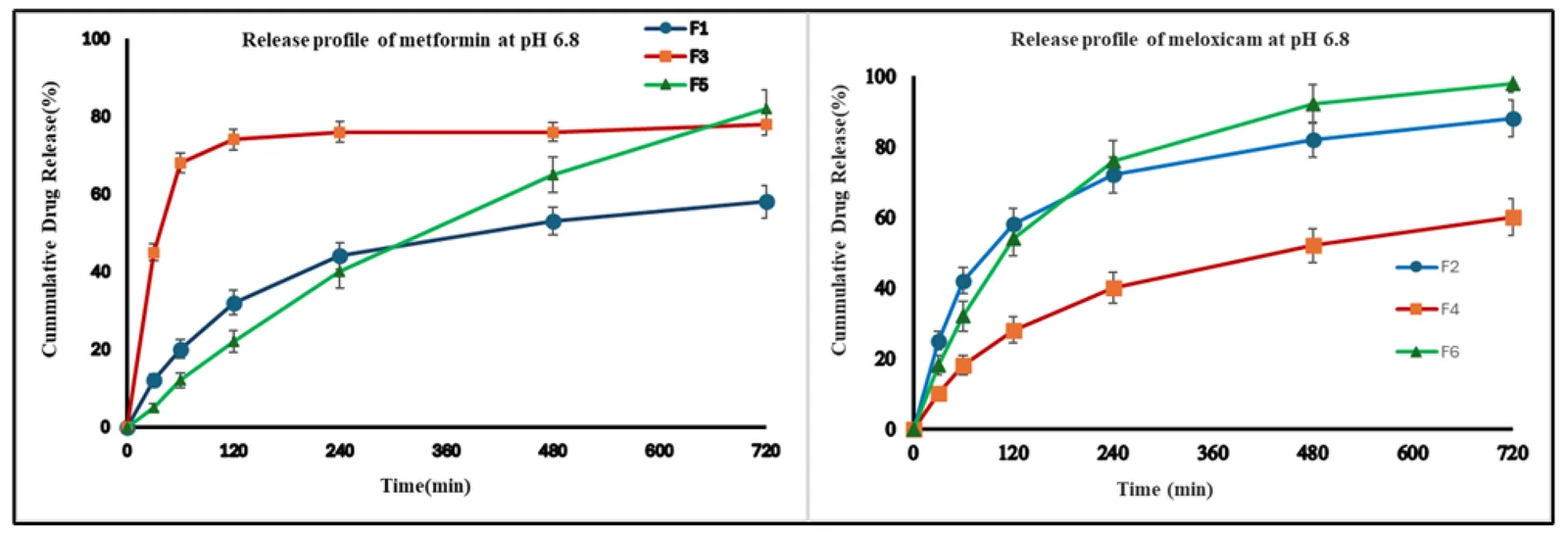
In vitro release profiles of metformin- and meloxicam-loaded formulations in simulated intestinal medium (pH 6.8). Data are presented as mean ± SD (*n* = 3). Similarity between selected dissolution profiles was evaluated using the similarity factor (f_2_) [34].

Meloxicam release was also very different at 15 min (F(2,6) = 191.83, *p* < 0.001) with F6 highest (62.0 ± 4.3%), F2 intermediate (28.0 ± 3.0%), F4 lowest (12.0 ± 1.8%). Meloxicam release in acidic conditions is a result of two factors: the structure of the carrier and the poor aqueous solubility of the drug at low pH [18]. Even after the CaCO_3_ carrier has dissolved, the released meloxicam must dissolve in the acidic medium and that is a solubility-limited step independent of the structure of the carrier [33], and the mechanism of a drug’s own solubility is the same in biopharmaceutical science: the drug can be expected to release slowly and in a time scale and is not inhibited by the structure of the carrier. F6 exhibits a more rapid release because the PVP modification preserved the highly porous vaterite structure, enhancing both structural stability and wettability. In contrast, F2 demonstrates a slower release rate because the native carrier lacks PVP-mediated stabilization, resulting in lower wettability and impeded drug diffusion within the acidic medium. The low F4 release is a consequence of both the vaterite transformation and the deteriorating structure of the vessel. Figure 8 shows the release profiles of metformin and meloxicam under simulated intestinal media (pH 6.8).

The release profiles at pH 6.8 were quite different from the ones we had observed at acidic pH. Since calcium carbonate is more stable at the pH of near neutrality [7], the diffusion of the drug through the carrier structure (as diffusion is generally controlled in porous solid matrices) and the drug distribution in the matrix and in the drug physical state became increasingly significant for the release [35].

Among the metformin formulations, F5 reached ~82% cumulative release of the drug at 720 min compared to 58% for F1 (f_2_ = 38.62), demonstrating dissimilar profiles, while F3 exhibited an initial burst release followed by a plateau. This high rate of release for F5 would be consistent with the preserved carrier structure and the reduced crystallinity of the drug that was already shown in previous studies. The burst and plateau behavior in F3 is consistent with the same high vaterite-to-calcite transformation that we have already observed in the previous studies.

The meloxicam formulations behaved differently under simulated intestinal conditions. F6 generated the highest cumulative release at 720 min (98%) compared to F2 (88%), while F4 exhibited the lowest (60%). Similarity factor analysis of F2 and F6 revealed f_2_ = 54.75 compared to F6 (i.e., f_2_ ≥ 50 similarity factors) [34], indicating that, despite the larger final release of F6, the two formulations had a similar overall dissolution pattern. This is consistent with the structural results: both the porous vaterite carrier of F2 and F6 were well preserved and diffusion pathways were preserved at near neutral pH. F4’s much lower release is due to the porous vaterite carrier being converted into the denser calcite carrier during long loading and therefore lower pore accessibility and less drug diffusion. The higher cumulative release of F6 relative to F2 is probably due to the higher structural stability, greater adsorption capacity and lower drug crystallinity due to the PVP modification of the vaterite carrier. This is also in line with recent evidence that polymer-mediated vaterite stabilization is associated with a more stable structure and a better performance in CaCO_3_-based drug delivery systems [36] and increases the overall release rate without changing the overall release pattern.

### 2.6. Release Kinetic Analysis

To further elucidate the drug release mechanisms, the dissolution data were fitted to zero-order, first-order, Higuchi, and Korsmeyer–Peppas kinetic models [36] (Table 1).

The Korsmeyer–Peppas model was applied only to the initial release stage (<60% cumulative release), consistent with the validity range established for this model, which also defines the release exponent (*n*) thresholds used to interpret the dominant transport mechanism [37].

The Korsmeyer–Peppas model was able to capture the release stage for all the formulations except for F3. In this case, the early drug release was mainly governed by diffusion. For different formulations, the release exponent varied, but diffusion and formulation-specific structural effects were equally important. F1 was characterized by classical Fickian diffusion (*n* = 0.492, in the Fickian range for spherical geometry) [35,37] in view of the preserved porous vaterite structure that provided an interconnected diffusion structure to support drug transport. F2, F4, and F6 had unusual (non-Fickian) transport [37]. This indicates that diffusion and formulation-dependent matrix effects were important for their release and that the porous vaterite structure is preserved in F2 and F6 to allow for diffusion and the diffusion pathway is not very wide (as opposed to diffusion and the diffusion of the drug).

For F5, however, it was not the case due to the denser calcite structure. Rather than diffusion alone, it is probably due to diffusion through the preserved porous carrier and PVP-mediated structural stabilization which kept the vaterite framework intact and produced a controlled release process. However, F3 could not be described in any of the models, suggesting that the release process is dominated by a burst of the drug, followed by a rapid depletion of the drug fraction due to extensive structure deterioration and loss of porosity. These results are also supported by the analysis of the dissolution profiles. F1 and F4 were more in agreement with the Higuchi model showing diffusion-driven release, while F2 and F5 were more in agreement with the first-order model showing a release rate that depends on the amount of drug in the carrier [35]. F6 was quite similar to both the first-order and Korsmeyer–Peppas model, and its release behavior is not understood in a single model, indicating that there are multiple transport processes in play.

However, the mechanism of drug release from the CaCO_3_ carriers developed cannot be explained by a single mathematical model; it is strongly dependent on the structure and process of the carrier and the way it is loaded. The porous structure of the vaterite-rich carriers was able to allow diffusion and drug release to occur in a controlled manner, but the loading strategy in the denser calcite polymorph led to pore connectivity and less drug release.

## 3. Materials and Methods

### 3.1. Materials

Metformin hydrochloride and meloxicam were kindly provided by Pioneer Co-Pharmaceutical Industries, Baghdad, Iraq. Calcium chloride dihydrate (CaCl_2_·2H_2_O), sodium carbonate (Na_2_CO_3_), ethylene glycol, ethanol, polyvinylpyrrolidone K30 (PVP-K30), and Tween 80 were purchased from Sigma-Aldrich, St. Louis, MO, USA, except where otherwise stated. All solvents were of analytical grade, and all chemicals were used as received. All experiments were performed in accordance with standard laboratory health and safety procedures.

### 3.2. Preparation of CaCO_3_-Based Carriers

**Figure 9:**
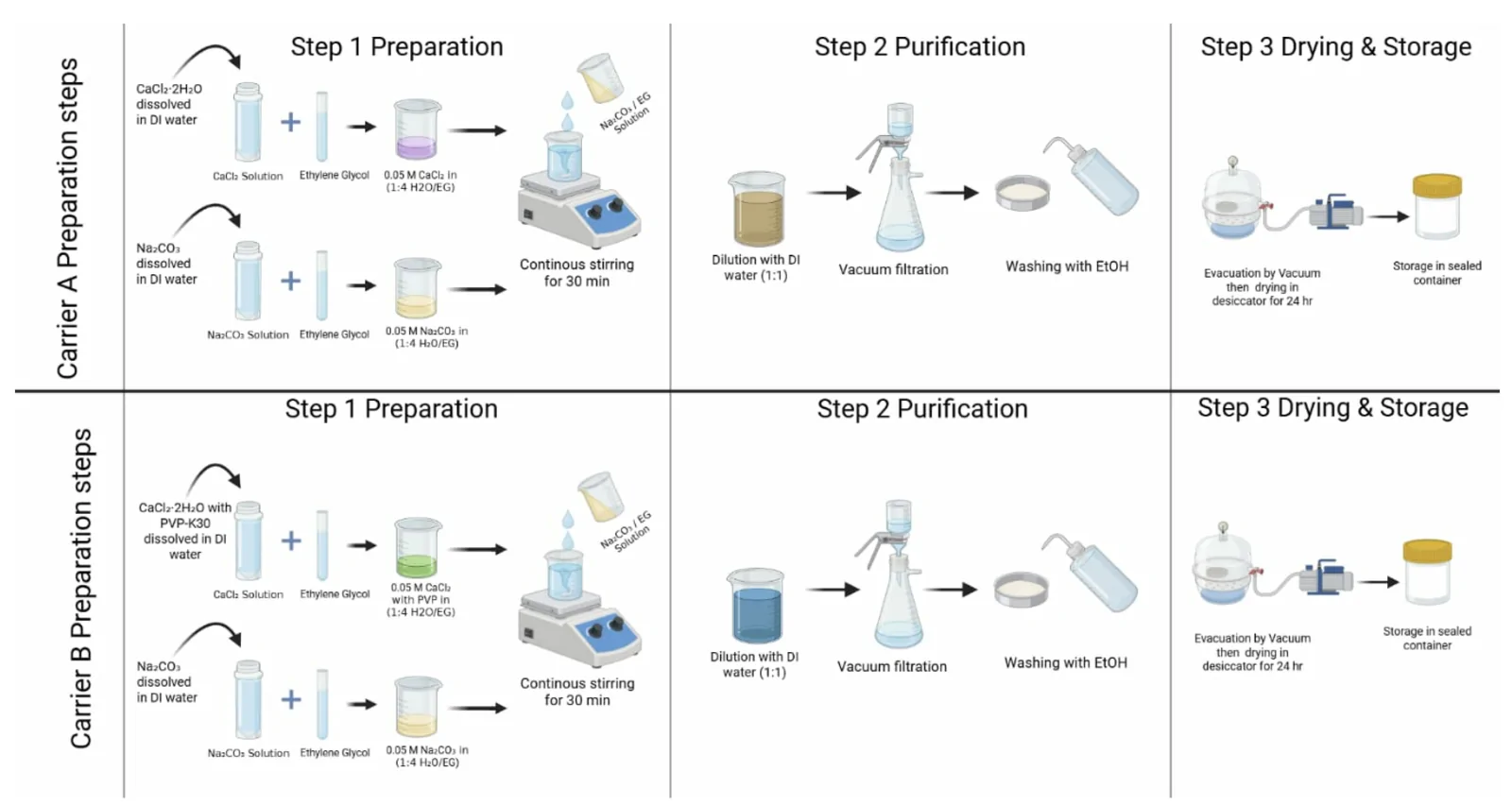
Schematic representation of preparation of CaCO_3_ carriers A and B (created using draw.io (JGraph Ltd., London, UK; https://www.drawio.com)).

We prepared two CaCO_3_-based carriers, a native carrier (Carrier A) and a PVP-K30-modified carrier (Carrier B). In Carrier A, 0.368 g of CaCl_2_·2H_2_O and 0.26 g of Na_2_CO_3_ were dissolved in 10 mL of deionized water. Each solution was then diluted with 40 mL of ethylene glycol under continuous magnetic stirring to have a final concentration of 0.05 M [38]. The Na_2_CO_3_ was added to the CaCl_2_ solution at a controlled rate of 10 mL/min under continuous stirring at 1000 rpm at 25 °C. The reaction mixture was stirred for 30 min to allow particles to mature. The resulting dispersion was diluted 1:1 with deionized water and vacuum-filtered through a 0.45 µm membrane filter. The recovered precipitate was washed three times with ethanol, vacuum-dried in a desiccator for 24 h, and stored in tightly sealed containers for use later on (Figure 9). Carrier B was also synthesized in the same manner except that 0.125 g of PVP-K30 was first dissolved in the CaCl_2_ solution before precipitation (Figure 9) [13].

### 3.3. Loading of Model Drugs

The carriers (A and B) were used as drug delivery platforms for two model therapeutic agents with different physicochemical properties: metformin, a hydrophilic drug, and meloxicam, a lipophilic drug. Drug loading strategies were classified as rapid loading methods (co-precipitation and sonication-assisted adsorption) and a conventional post-synthetic adsorption method (slow loading). Six formulations (F1–F6) were prepared as described below [32].

### 3.4. Co-Precipitation Loading (F1 and F5)

Formulation F1 was prepared via the co-precipitation method, where 25 mg of metformin was dissolved in the CaCl_2_ precursor solution before particle synthesis, and the same preparation procedure was followed for Carrier A. Formulation F5 was also done using the same method except that Carrier A was replaced by PVP-K30-modified Carrier B [7].

#### 3.4.1. Sonication-Assisted Adsorption Loading (F2 and F6)

Formulations F2 and F6 were developed to add meloxicam into Carrier A and Carrier B with the sonication-assisted adsorption strategy [39]. In short, 100 mg of each carrier was dispersed in 50 mL of deionized water with 0.25 mL of Tween 80 as a dispersing agent. Moreover, 10 mg of meloxicam was dissolved in 2 mL ethanol and then added to the carrier dispersion. After an initial 5 min of equilibration with continuous stirring, the suspension was probe sonicated for 3 min at 40% amplitude (30 s on/10 s off) in pulsed mode. In order to minimize the effects of thermally induced physicochemical changes during sonication, the sample vessel was kept in an ice bath [39]. After this, the dispersion was magnetically stirred for 4 h to ensure adsorption equilibrium. The drug-loaded particles were recovered by vacuum filtration, vacuum dried, kept in a desiccator for 24 h, and placed in sealed containers for further analysis.

#### 3.4.2. Post-Synthetic Adsorption Loading (F3 and F4)

The post-synthetic adsorption strategy was applied for the production of F3 and F4 in the following 12 h loading time, with Carrier A used as the adsorption matrix [40]. In F3, 100 mg of Carrier A was added to 50 mL of deionized water and 25 mg of metformin in place, and the suspension was kept under continuous magnetic stirring for 12 h to reach adsorption equilibrium. The dispersion was vacuum-filtered, and the recovered solid was vacuum-dried in a desiccator for 24 h and stored in sealed containers for analysis. In F4, the same procedure was applied, but 10 mg of meloxicam was added in 2 mL of ethanol instead of metformin. The complete composition and operational parameters of all formulations are described in Table 2.

### 3.5. Characterization of CaCO_3_ Carriers

#### 3.5.1. Morphological Characterization of CaCO_3_

We used field emission scanning electron microscopy (FE-SEM) to characterize the shape and surface of the carrier formed using CaCO_3_ (Carrier A and Carrier B) and their drug-loaded versions (F1–F6) [41]. We used an FE-SEM (Inspect F50, FEI Company, Hillsboro, OR, USA) with an accelerating voltage of 30 kV, and powdered samples were placed on aluminum stubs with double-sided conductive carbon tape. The samples were then sputter-coated with a thin gold layer by an automated coater (PELCO SC-7, Ted Pella, Inc., Redding, CA, USA) to enhance the electrical conductivity and minimize the charging artifacts. The microstructural examination was conducted at varying magnifications to evaluate particle architecture and surface topography. To generate number-weighted particle size distributions, morphometric image analysis and spatial scaling of the FE-SEM micrographs were performed using Fiji software (ImageJ version 1.54t, 16 May 2026, NIH, Bethesda, MD, USA)to calculate the mean diameter and standard deviation for each carrier and loaded formulation.

#### 3.5.2. Comparative Accessible Adsorption Capacity

The accessible adsorption capacity of the synthesized carriers was indirectly measured with methylene blue (MB) adsorption [42]. A stock MB solution (100 mg/L) was prepared in ultrapure water, and a calibration curve (0–10 mg/L, R^2^ > 0.99) was established at 664 nm with phosphate-buffered saline (PBS, 10 mM, pH 7.4). In the adsorption test, 20 mg of each sample (Carrier A and Carrier B) were dispersed in 50 mL of MB solution (100 mg/L in PBS, pH 7.4) and incubated under orbital shaking at 150 rpm for 2 h at 25 °C to reach adsorption equilibrium. 10 mL aliquots were centrifuged at 6000 rpm for 10 min to sediment the particles. A 1 mL aliquot of the supernatant was diluted with MB-free PBS, and the absorbance of the samples was measured at 664 nm. The equilibrium adsorption capacity (qe, mg/g) was calculated as follows:

$$q_{e}=\frac{\left(C_{initial}-C_{final}\right)\times V}{m}$$

where *C*_*initial*_ is the initial MB concentration (100 mg/L), *C*_*final*_ is the equilibrium concentration determined from the calibration curve, *V* is the total solution volume (0.05 L), and *m* is the dry mass of the adsorbent (0.02 g).

#### 3.5.3. Zeta Potential Measurements

The zeta potential of the synthesized carriers (Carrier A and Carrier B) and the drug-loaded formulations (F1–F6) was measured using a Malvern Zetasizer Nano ZS (Malvern Instruments, Malvern, UK) with a disposable folded capillary cell (DTS1070) [43]. A sample of 10 mg was weighed and dispersed in 10 mL of deionized water and diluted as needed to obtain a homogeneous and stable dispersion for comparison.

#### 3.5.4. XRD Diffraction Study (PXRD)

Powder X-ray diffractometer (Malvern Panalytical, Malvern, Worcestershire, UK) (PXRD) analysis was performed to characterize the crystalline structure of the synthesized carriers (Carrier A and Carrier B), the drug-loaded formulations, and the pure model drugs for comparative phase identification [20]. Data were measured using an Aeris powder X-ray diffractometer (Malvern Panalytical, Malvern, Worcestershire, UK) equipped with a PIXcel1D-Medipix3 detector and a Cu Kα radiation source. In the θ–2θ scanning mode, the diffraction was scanned in a 5–100° angular range.

#### 3.5.5. FTIR Determination

Fourier-transform infrared (FTIR) spectroscopy was performed using a Nicolet iS10 FTIR spectrometer (Thermo Scientific, Waltham, MA, USA) to evaluate the chemical properties and potential molecular interactions of the pure drugs (metformin and meloxicam), the blank carriers (Carrier A and Carrier B), and the drug-loaded formulations (F1–F6) [44] and is available under the guidance of T1-F6 spectra. For analysis, samples (1–2 mg) were finely ground, thoroughly mixed with potassium bromide (KBr), and compressed into pellets. FTIR spectra were recorded over the range 4000–400 cm^−1^.

#### 3.5.6. Differential Scanning Calorimetry (DSC)

Differential scanning calorimetry (DSC) analysis was conducted to evaluate the thermal behavior of the synthesized carriers (Carrier A and Carrier B), the corresponding drug-loaded formulations, and the pure model drugs [45]. Measurements were performed using a Shimadzu DSC-60 instrument (Kyoto, Japan). Accurately weighed samples (2–4 mg) were placed in sealed aluminum pans and heated from ambient temperature to 600 °C at a rate of 10 °C/min. The instrument was calibrated with indium as the reference standard, and nitrogen was used as the purge gas at a flow rate of 30 mL/min throughout the analysis.

#### 3.5.7. Encapsulation Efficiency

Encapsulation efficiency (EE%) was determined by measuring the drug recovered from the drug-loaded particles after the carrier matrix of CaCO_3_ had completely dissolved [46]. The drug-loaded particles were dispersed in 10 mL of 0.1 N HCl to dissolve the carrier matrix and weighed accordingly. For meloxicam, ethanol was added before filtration to make sure that the drug had completely solubilized. The solutions were filtered through a 0.2 µm membrane filter and diluted with the corresponding analytical medium. The drug content was measured using a UV–visible spectrophotometer (UV-1650PC, Shimadzu, Kyoto, Japan) at 233 nm for metformin and 365 nm for meloxicam. Independent calibration curves were constructed for each specific analytical medium to account for any solvent-induced spectral shifts. Encapsulation efficiency was calculated as:

$$EE\%=\frac{Weight\;encapsulated}{weight\;initial}\times 100\%$$

#### 3.5.8. In Vitro Release Study

In vitro drug release of the drug-loaded formulations was studied with a USP type II (708-DS, Agilent Technologies, Santa Clara, CA, USA) dissolution apparatus (paddle method). A dialysis membrane bag (molecular weight cut-off: 12 kDa) was soaked overnight in the corresponding dissolution medium to ensure complete hydration before use [47]. For the metformin-loaded formulations (F1, F3, F5), an appropriate amount of each formulation (25 mg of metformin based on the encapsulation efficiency previously determined) was placed inside the dialysis bag so that it was safely sealed and immersed in 500 mL of the dissolution medium (0.1 N HCl, pH 1.2 or phosphate buffer, pH 6.8). The dissolution study was performed at 37 ± 0.5 °C with the paddle rotating at 100 rpm. At specified intervals (5, 15, 30, 60, and 120 min for 0.1 N HCl, pH 1.2; and 30, 60, 120, 240, 480, and 720 min for phosphate buffer, pH 6.8), 5 mL aliquots were removed and replaced with an equal volume of fresh pre-warmed medium to maintain sink conditions. Drug concentration was measured with a UV-visible spectrophotometer (Model UV-1800, Shimadzo, Kyoto Japan) at 233 nm.

For the meloxicam-loaded formulations (F2, F4, and F6), the same process was followed except 1% *w/v* sodium lauryl sulfate (SLS) was added to the same acid medium to maintain the sink [48] and the released drugs were quantified at their respective pH-dependent absorption maxima: 351 nm in 0.1 N HCl (pH 1.2) and 362 nm in phosphate buffer (pH 6.8). Independent calibration curves were constructed and validated in each specific buffer to ensure accurate quantification and account for the pH-induced shifts in the meloxicam absorption spectrum. The release profiles of metformin and meloxicam-loaded formulations were fit with zero-order, first-order, Higuchi, and Korsmeyer–Peppas kinetic models of the drug release mechanism [49].

### 3.6. Statistical Analysis

All experiments were performed using three independent synthesis batches for each formulation (*n* = 3) to accurately capture the reproducibility of the preparation methods, and the resulting data are recorded as the mean ± standard deviation (SD) across these independent batches. Data analysis was conducted using OriginPro 2024 (OriginLab, Northampton, MA, USA). Depending on the data set, the differences between groups were evaluated in a Student’s t-test or ANOVA. The ANOVA was followed by Tukey’s post hoc test to detect specific differences in the groups. The in vitro dissolution profiles in the simulated intestinal medium (pH 6.8) were then compared using the similarity factor (f_2_). For all analyses, a p-value of less than 0.05 was considered to be significant.

## 4. Conclusions

This study has shown that the composition and the loading strategy are crucial factors in the structure and drug delivery of porous CaCO_3_ carriers. A comparison of metformin and meloxicam showed that the drug physicochemical properties are a major factor for the loading strategy and therefore the loading strategy should be more sensitive to the drug. We observed that the porous vaterite-rich structure was the best candidate for a larger adsorption potential, better encapsulation efficiency, lower drug crystallinity and drug release, while the change into the denser calcite polymorph had negative effects on the carrier performance. The PVP modification stabilized the vaterite-rich structure, gave more adsorption capacity and drug loading, and the overall release performance was better. A kinetic analysis of the initial release showed that it was largely diffusion controlled, while the release behavior was influenced by the structure and formulation strategy. Our results provide a comparative evaluation of six distinct preparations, demonstrating how specific combinations of carrier composition and loading strategy affect performance. These findings suggest that optimally prepared PVP-stabilized porous vaterite CaCO_3_ formulations serve as viable platforms for targeted drug delivery. In future studies, direct porosity profiling (e.g., BET nitrogen physisorption), biorelevant dissolution testing, and in vivo evaluation will be strictly necessary to further confirm the internal pore dynamics and translational potential of this delivery platform [50,51].

## Figures and Tables

**Table 1:** Mathematical modeling of in vitro drug release kinetics for the developed CaCO_3_-based formulations.

Formulation	Zero-Order (R^2^)	First-Order (R^2^)	Higuchi (R^2^)	Korsmeyer–Peppas (R^2^)	*n*
F1	0.806	0.884	0.9624	0.957	0.492
F3	0.382	0.465	0.519	—	—
F5	0.973	0.9972	0.9734	0.990	0.987
F2	0.7108	0.9187	0.9163	0.982	0.6071
F4	0.875	0.945	0.9878	0.974	0.551
F6	0.8016	0.9979	0.9555	0.999	0.7992

**Table 2:** Composition and drug-loading strategies of the developed CaCO_3_-based formulations.

Formulation	Carrier Type	Model Drug	Loading Strategy
F1	Carrier A (native CaCO_3_)	Metformin	Co-precipitation
F2	Carrier A (native CaCO_3_)	Meloxicam	Sonication-assisted adsorption
F3	Carrier A (native CaCO_3_)	Metformin	Post-synthetic adsorption
F4	Carrier A (native CaCO_3_)	Meloxicam	Post-synthetic adsorption
F5	Carrier B (PVP-K30-modified CaCO_3_)	Metformin	Co-precipitation
F6	Carrier B (PVP-K30-modified CaCO_3_)	Meloxicam	Sonication-assisted adsorption

## Data Availability

The original contributions presented in this study are included in the article. Further inquiries can be directed to the corresponding author.
